# Chondroprotective Effects and Multitarget Mechanisms of Fu Yuan Capsule in a Rat Osteoarthritis Model

**DOI:** 10.1155/2017/8985623

**Published:** 2017-01-11

**Authors:** Li Zeng, Cai Zhi Xiao, Zi Ting Deng, Rong Heng Li

**Affiliations:** ^1^Department of Combination of Chinese and Western Medicine, The First Affiliated Hospital of Chongqing Medical University, Chongqing 400016, China; ^2^Laboratory Research Center, The First Affiliated Hospital of Chongqing Medical University, Chongqing 400016, China; ^3^Department of Traditional Chinese Medicine for Tumor, Chongqing Cancer Institute, Chongqing 400030, China; ^4^Department of Rehabilitation Medicine, The Affiliated Rehabilitation Hospital of Chongqing Medical University, Chongqing 400016, China

## Abstract

Fu Yuan Capsule (FYC) has been clinically used for osteoarthritis (OA) and its related diseases for many years in China. However, its pharmacological mechanism remains unclear. This study aimed to investigate the potential chondroprotective effects of FYC on articular cartilage. Rat OA model was induced by anterior cruciate ligament transection. A group of rats was treated with FYC for 12 weeks. Joint structure, types I and II collagen, and proteoglycan were evaluated by histological examination. The expression of C-terminal crosslinking telopeptide of type II collagen, hydroxyproline, a disintegrin and metalloproteinase with thrombospondin motifs, matrix metalloproteinase, interleukin-1 beta, nitric oxide, prostaglandin E2, heat-shock protein 70, transforming growth factor-beta, osteoprotegerin, and receptor activator of nuclear factor *κ*B ligand were detected. Treatment with FYC could protect against articular cartilage injury. FYC treatment significantly decreased the extracellular matrix degradation factors and inflammatory mediators. Moreover, articular cartilage protective factors were increased in the FYC group. The current finding suggests that FYC protects articular cartilage in a rat OA model through various ways. Thus, it may be an effective agent for OA treatment.

## 1. Introduction

Osteoarthritis (OA) has become a significant clinical problem worldwide and is expected to worsen with the growth of the aging population. Existing drug treatments include nonsteroidal anti-inflammatory drugs and selective cyclooxygenase-2 inhibitors. However, pharmacological interventions do not exceed beyond addressing chronic pain and fail to prevent cartilage damage and the associated destruction of other joint tissues. Therefore, safer and better tolerated agents are needed for OA treatment.

In recent years, interest in OA treatment with plant-based therapy, including traditional Chinese medicine, has been increasing. Generally, a traditional Chinese medicine contains multiple active ingredients that aim at multiple targets [1]. An excellent example of this formula is the Fu Yuan Capsule (FYC), which is against OA and its related diseases. Our previous studies showed that FYC is an effective drug for knee OA patients [2]. However, the pharmacological mechanism of FYC remains unclear.

A growing body of evidence demonstrates that the extracellular matrix (ECM) degradation factors, inflammatory mediators, and articular cartilage protective factors are all significantly changed in OA. Moreover, a disintegrin and metalloproteinase with thrombospondin motifs (ADAMTS), matrix metalloproteinase (MMP), interleukin-1 beta (IL-1*β*), nitric oxide (NO), prostaglandin E2 (PGE2), heat-shock protein 70 (HSP70), transforming growth factor-beta (TGF-*β*), osteoprotegerin (OPG), and receptor activator of nuclear factor *κ*B ligand (RANKL) play an important role based on their ability to destruct or protect articular cartilage [3–6]. In the present study, the effects of FYC on ECM in a rat OA model were investigated. The aforementioned factors possibly involved in the process of OA were specifically assayed.

## 2. Materials and Methods

### 2.1. Animals and Drug Administration

Forty-five SD rats were obtained from the Animal Center of Chongqing Medical University (Chongqing, China) (certificate number SCXK [Yu] 2007-0001). FYC was mainly composed of* Epimedium*,* Ginseng*, Radix Astragali, Pilose antler, Medlar, and Radix Notoginseng. It was produced by Xi Er An Pharmaceutical Company (Chongqing, China). Each capsule weighed 0.41 g, which is equivalent to 3 g of crude drug. The research preparation approval number for this drug is [2005] 12 B-55. With the approval of the Institutional Animal Care and Use Committee of Chongqing Medical University, thirty rats underwent anterior cruciate ligament transection in the right posterior knee joint to generate an experimental model of OA as previously described [7]. These rats were then randomly divided into two groups. After two weeks, the rats received daily gavage of saline with or without FYC (0.3 mL, 2.26 g/kg·d) for twelve weeks. The remaining fifteen rats were used as controls.

### 2.2. Histologic Evaluations

Femoral epicondyles were removed, fixed in 4% paraformaldehyde, then decalcified, and processed through a series of increasing ethanol concentrations for dehydration. These samples were subsequently paraffin-embedded and sectioned at 4 *μ*m. The sections were then stained with hematoxylin and eosin for general histological assessment, where the grading of hematoxylin and eosin staining was determined according to Mankin's scoring system [8], or with Masson staining for assessment of cartilage collagen and toluidine blue for assessment of cartilage proteoglycan. For the immunohistochemical analysis of anti-rat primary polyclonal antibodies, slides were incubated with types I and II collagen antibodies (Boster Biological Engineering Co., Ltd., Wuhan, China) overnight at 4°C in a wet room and then with the secondary antibody (Boster Biological Engineering Co., Ltd., Wuhan, China). The expression of types I and II collagen was visualized by chromogen 3,3-diaminobenzidine immunolabeling. Finally, the sections were counterstained with hematoxylin. The contents of collagen and proteoglycan were quantitatively analyzed with Image-Pro Plus 7.0.

### 2.3. Sample Hydrolysis Method, Nitrate Reductase Method, and Enzyme-Linked Immunosorbent Assay (ELISA)

Hydroxyproline and C-terminal crosslinking telopeptides of type II collagen (CTX-II) are markers of collagen synthesis and degradation. In this study, these markers were measured using sample hydrolysis method kits (Jiancheng Biological Engineering Research Institute, Nanjing, China) and ELISA kits (Hua Mei Biotechnology, Wuhan, China) according to the manufacturer's instructions. ELISA kit for IL-1*β*, PGE2, and NO assay kit (nitrate reductase method) were purchased from Shengbo Technology Co., Ltd. (Chongqing, China) and Jiancheng Biological Engineering Research Institute (Nanjing, China), respectively.

### 2.4. Quantitative Real-Time Polymerase Chain Reaction

Total RNA was extracted using TRIzol reagent (Invitrogen, Shanghai, China) according to the manufacturer's instructions. The reverse transcriptase cDNA synthesis kit (TOYOBO, Japan) was used to obtain cDNA. The 2^−ΔΔCT^ method was performed to calculate the relative fold changes in mRNA expression. The sequences of the sense and antisense primers used for amplification are listed in Table 1.

### 2.5. Western Blot Analysis

Proteins were isolated using an extraction kit (Beyotime Institute of Biotechnology, Jiangsu, China). The protein extracts were resolved by sodium dodecyl sulfate-polyacrylamide gel electrophoresis, transferred onto PVDF membranes, and probed with primary antibodies overnight at 4°C. MMP-1, MMP-3, MMP-13, and *β*-actin antibodies were purchased from Proteintech Group (Man, UK). MMP-2, MMP-9, ADAMTS-4, and ADAMTS-5 antibodies were obtained from Santa Cruz (USA). HSP70 antibody was purchased from Boster Biological Engineering Co., Ltd. (Wuhan, China). The membranes were washed with TBS containing 0.05% Tween 20 and incubated with the appropriate HPR-linked secondary antibodies at 37°C for 1 h, followed by visualization using an enhanced chemiluminescence kit (Beyotime Institute of Biotechnology, Jiangsu, China).

### 2.6. Statistical Analysis

Data are expressed as mean ± standard deviation. Comparisons were made using one-way analysis of variance with SPSS version 18.0 for Windows (SPSS Inc., Chicago, IL, USA). A *p* value less than 0.05 was considered significant.

## 3. Results

### 3.1. Effects of FYC on Joint Structure

**Figure 1:**
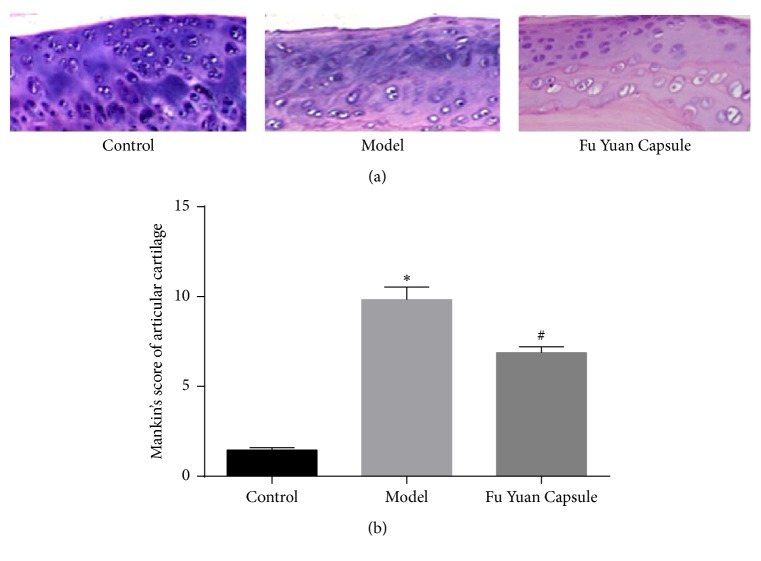
Histological analysis of articular cartilage sections. (a) Hematoxylin-eosin staining of knee joint sections in the control group showed a smooth surface and a clear laminar structure in the articular cartilage. By contrast, the cartilage surface in the model group was rough with some superficial leakage and ulcers. Furthermore, Fu Yuan Capsule-treated cartilage showed fewer lesions compared with the model group (magnification, 200x). (b) Mankin's score of articular cartilage. Data are expressed as mean ± SD. ^*∗*^*p* < 0.05 compared with the control group; ^#^*p* < 0.05 compared with the other groups.

The effect of FYC on the rat OA model was confirmed by histological analyses.  Figure 1(a) reveals that the model group exhibited some structural changes (superficial leakage and ulcers) in the articular cartilage compared with the control group. Moreover, treatment with FYC was associated with less damage in the cartilage compared with the model group.  Figure 1(b) shows the histological scores associated with the histological studies.

### 3.2. Effects of FYC on ECM

**Figure 2:**
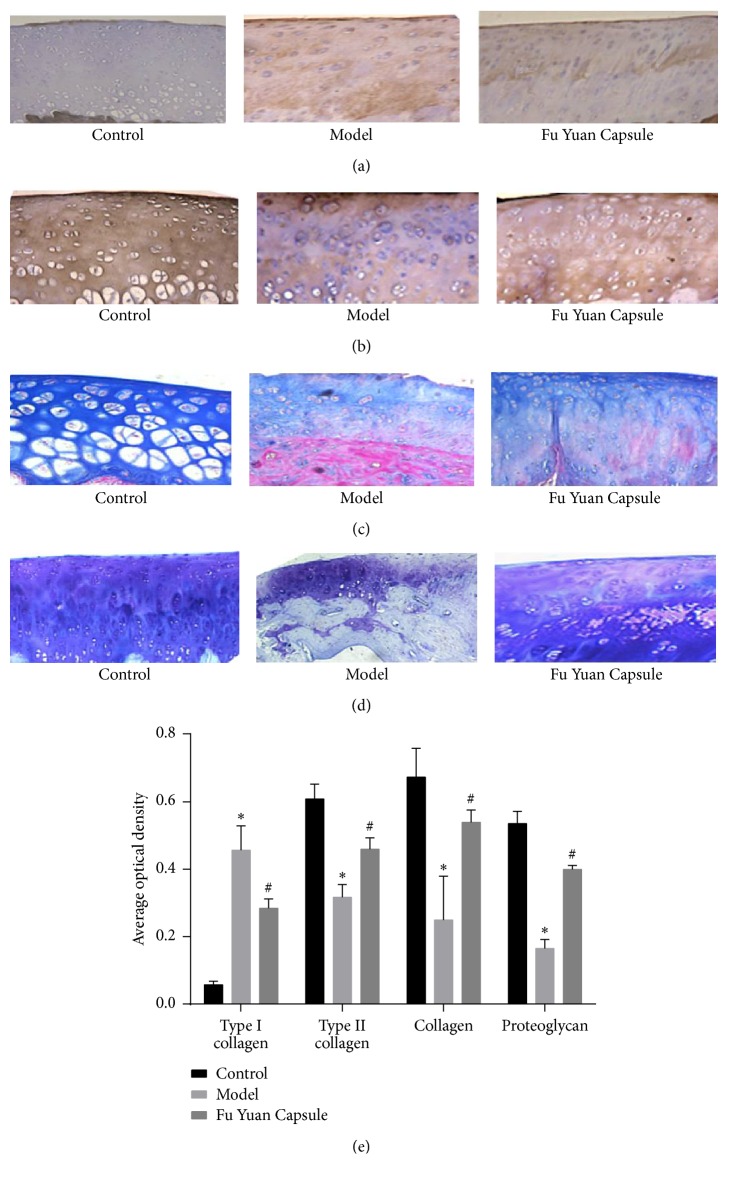
Effects of Fu Yuan Capsule on extracellular matrix in a rat osteoarthritis model. (a) Immunohistochemical staining of type I collagen (magnification, 200x). (b) Immunohistochemical staining of type II collagen (magnification, 200x). (c) Masson staining of collagen (magnification, 200x). (d) Toluidine blue staining of proteoglycan (magnification, 200x). (e) Average optical density of staining was calculated using image analysis software Image-Pro Plus 7.0. Data are expressed as mean ± SD. ^*∗*^*p* < 0.05 compared with the control group; ^#^*p* < 0.05 compared with the other groups.

**Figure 3:**
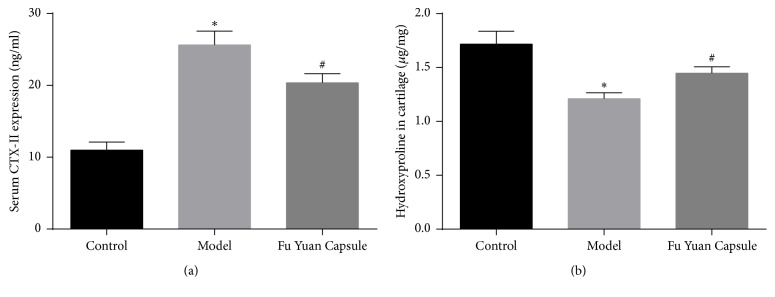
Effects of Fu Yuan Capsule on CTX-II and hydroxyproline in a rat osteoarthritis model. (a) ELISA detected CTX-II in serum. (b) Hydroxyproline was measured by a sample hydrolysis method. Data are expressed as mean ± SD (each group, *n* = 3). ^*∗*^*p* < 0.05 compared with the control group; ^#^*p* < 0.05 compared with the other groups.

Types I (Figure 2(a)) and II collagen (Figure 2(b)), collagen (Figure 2(c)), proteoglycan (Figure 2(c)), CTX-II, and hydroxyproline (Figure 3) were determined to identify the effect of FYC on ECM in the rat OA model. The results showed that the model group had significantly higher levels of type I collagen and CTX-II but lower levels of type II collagen, collagen, proteoglycan, and hydroxyproline as compared with the control group. Compared with the model group, the FYC group showed significantly decreased type I collagen and CTX-II levels and increased type II collagen, collagen, proteoglycan, and hydroxyproline levels.

### 3.3. Effect of FYC on ECM Degradation Factors

**Figure 4:**
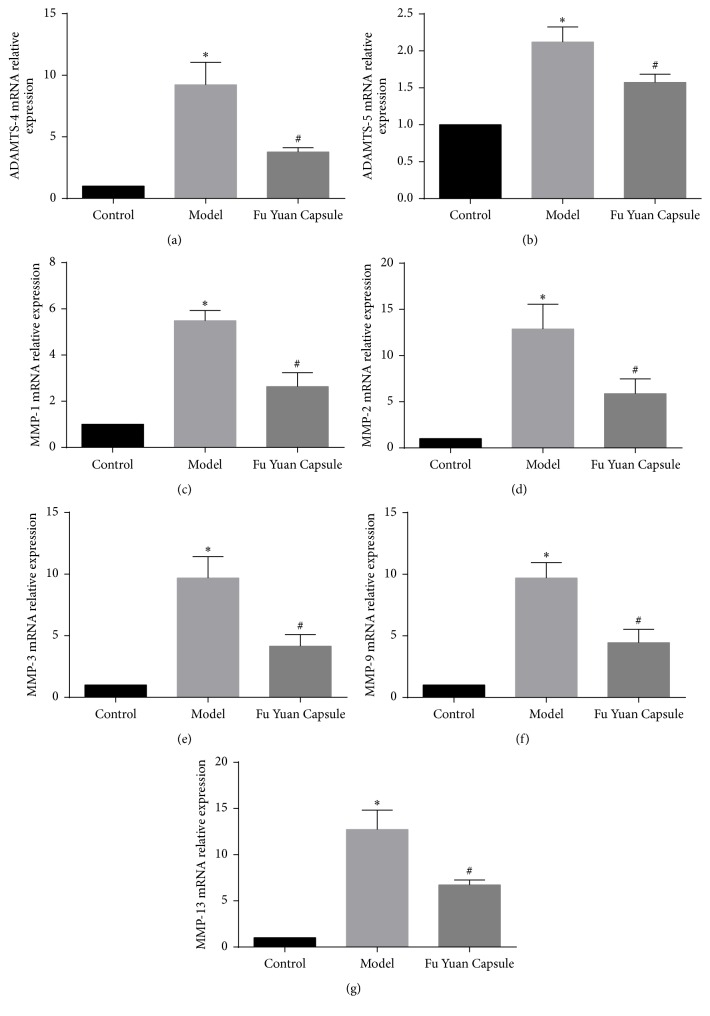
Effects of Fu Yuan Capsule on mRNA expression of extracellular matrix degradation factors. (a), (b), (c), (d), (e), (f), and (g), respectively, showed that ADAMTS-4, ADAMTS-5, MMP-1, MMP-2, MMP-3, MMP-9, and MMP-13 mRNA were detected by quantitative real-time polymerase chain reaction. Data are expressed as mean ± SD (each group, *n* = 3). ^*∗*^*p* < 0.05 compared with the control group; ^#^*p* < 0.05 compared with the other groups.

**Figure 5:**
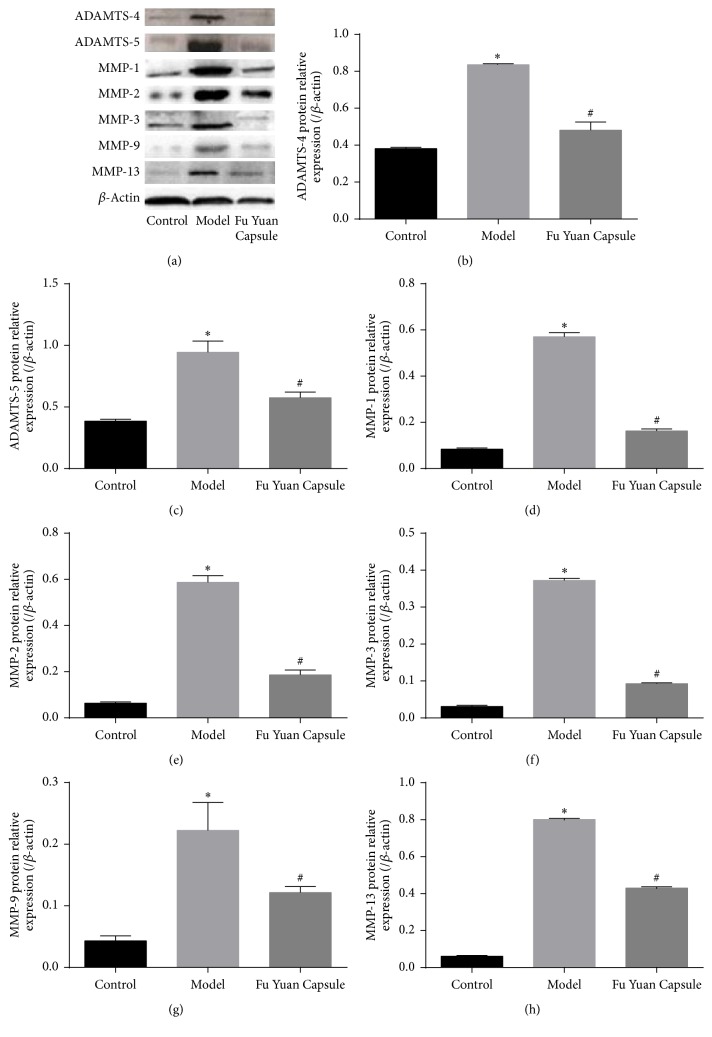
Effects of Fu Yuan Capsule on protein expression of extracellular matrix degradation factors. Representative Western blotting bands (a) and semiquantitative analysis of ADAMTS-4 (b), ADAMTS-5 (c), MMP-1 (d), MMP-2 (e), MMP-3 (f), MMP-9 (g), and MMP-13 (h) in various groups. (a) Each row represents images cropped from different gels probed with different antibodies. Data are expressed as mean ± SD (each group, *n* = 3). ^*∗*^*p* < 0.05 compared with the control group; ^#^*p* < 0.05 compared with the other groups.

To determine ECM degradation factors under different conditions, we measured the expression of ADAMTS-4, ADAMTS-5, MMP-1, MMP-2, MMP-3, MMP-9, and MMP-13 in OA cartilage (Figures 4 and 5). Compared with the control group, the model group showed significantly increased ECM degradation factors. The increased factors in the model group were restrained by FYC.

### 3.4. Effect of FYC on Inflammatory Mediators

**Figure 6:**
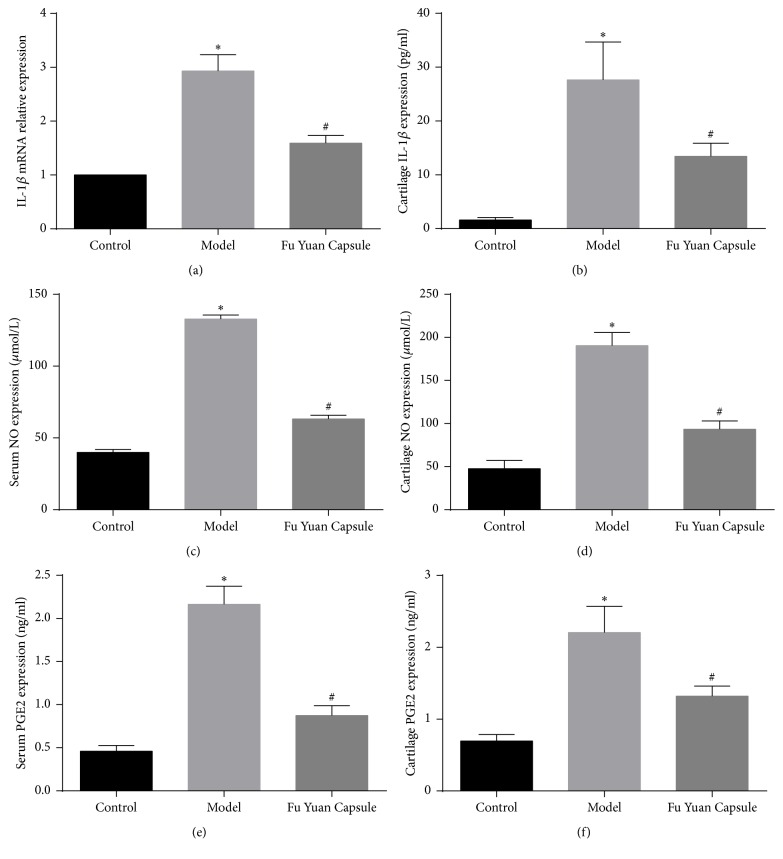
Effects of Fu Yuan Capsule on inflammatory mediators. IL-1*β* mRNA (a) was detected by quantitative real-time polymerase chain reaction. The expression of NO (c) and PGE2 (e) was detected by enzyme-linked immunosorbent assay in serum. The expression of IL-1*β* (b), NO (d), and PGE2 (f) was detected by enzyme-linked immunosorbent assay in cartilage. Data are expressed as mean ± SD (each group, *n* = 3). ^*∗*^*p* < 0.05 compared with the control group; ^#^*p* < 0.05 compared with the other groups.

IL-1*β*, NO, and PGE2 are important inflammatory mediators in OA. They were significantly increased in the model group as compared with those in the control group. Likewise, increased inflammatory mediators were reversed by FYC (Figure 6).

### 3.5. Effect of FYC on Articular Cartilage Protective Factor

**Figure 7:**
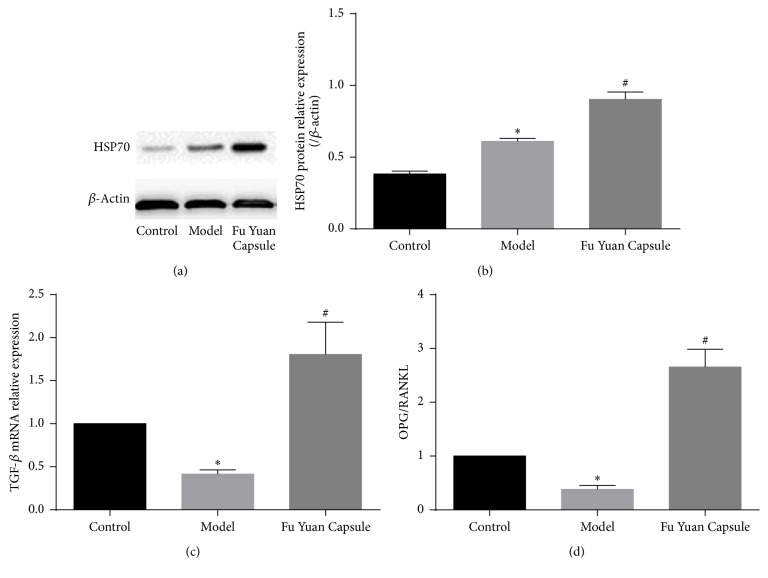
Effects of Fu Yuan Capsule on articular cartilage protective factor. Representative Western blotting bands (a) and semiquantitative analysis of HSP70 (b) in various groups. TGF-*β* mRNA (c) was detected by quantitative real-time polymerase chain reaction. Quantitative real-time polymerase chain reaction analysis was also performed to examine the mRNA expression of OPG and RANKL (d). Data are expressed as mean ± SD (each group, *n* = 3). ^*∗*^*p* < 0.05 compared with the control group; ^#^*p* < 0.05 compared with the other groups.

TGF-*β*, HSP70, and OPG play an important role in articular cartilage protection. As shown in Figure 7, the extent of TGF-*β*, HSP70, and OPG/RANKL ratio was significantly increased as compared with that in the model and control groups.

## 4. Discussion

OA is a heterogeneous, complicated joint disorder whose pathogenesis is affected by multiple etiologies, including aging and obesity, as well as by mechanical, biochemical, and genetic factors [9]. Traditional Chinese medicine has fought against OA and its related diseases for many years in China. Our research group has been focusing on the role of FYC in OA. In our previous clinical research, the effectiveness and safety of FYC for OA were identified based on an observational clinical research [2]. In the present study, we analyzed the effect of FYC on collagen and proteoglycan, including ADAMTS, MMPs, IL-1*β*, NO, PGE2, TGF-*β*, HSP70, OPG, and RANKL levels, which are involved in ECM degradation, as well as articular cartilage apoptotic and articular cartilage protective pathways. The experimental results showed that FYC could effectively protect ECM, reduce ECM degradation factors, depress inflammatory mediators, and increase articular cartilage protective factor in a rat OA model.

Articular cartilage degradation is the most predominant change observed in OA. To our knowledge, this study is the first to use a variety of methods to detect the effect of FYC on collagen and proteoglycan. Proteoglycans and type II collagen are the major constituents of cartilage. Type II collagen is localized almost exclusively in cartilage. Thus, measurements of fragments derived from this protein may potentially represent a specific marker for cartilage degradation. Previous studies reported that the concentrations of CTX-II in urine and serum reveal the extent of cartilage damage and are a good marker of OA progression [10–13]. The FYC could protect the collagen and proteoglycan of the articular cartilage in a rat OA model in the current study. This result is consistent with that in the rabbit OA model in our previous study.* Epimedium* and* Ginseng* are the major components of FYC. Other studies found that they have a protective effect on articular cartilage in OA [14, 15]. This finding also supports our results from another perspective.

The mechanistic details of OA pathogenesis remain to be elucidated. ECM degradation is an important contributing factor in articular cartilage injury. The ADAMTS family is known to influence the development, angiogenesis, coagulation, and progression of arthritis. Among the 19 members of ADAMTS family, ADAMTS-4 and ADAMTS-5 have received significant attention in the pathology of arthritic joint diseases because they are the most efficient aggrecanases in vitro. Several experiments have demonstrated that neutralizing antibodies and other methods play a significant role in the models of arthritis in restraining ADAMTS-4 or ADAMTS-5 [3]. ADAMTS are the main proteinases responsible for aggrecan cleavage in the early events of cartilage remodeling. The MMPs start participating in this process during the development of the disease and continue with collagen degradation. MMP-1, MMP-3, and MMP-13 are more restricted to cartilage and drive OA progression; they not only target the collagens in cartilage for degradation, but also degrade proteoglycan, osteonectin, and perlecan in cartilage [16]. In previous decades, numerous MMP inhibitors have been suggested as candidates for OA treatment. The results in this study are consistent with a role for these factors in regulating articular cartilage in OA. Moreover, the present results demonstrated that the protective effect of FYC on ECM was partially achieved by inhibiting ADAMTS-4, ADAMTS-5, MMP-1, MMP-2, MMP-3, MMP-9, and MMP-13.

The past decade has seen a gradual shift in our understanding of the mechanisms underlying OA. We now know that this disease affects the entire joint structure, and inflammation has a critical role in its pathogenesis [17]. IL-1*β* is a pleiotropic proinflammatory cytokine that plays a significant role in the development of OA by stimulating several mediators that contribute to cartilage degradation. NO and PGE2 are important inflammatory mediators in OA pathogenesis. Overproductions of NO and PGE2 are correlated to the pathophysiology of OA. IL-1*β* could significantly increase the production of NO and PGE2. Some potential agents in OA treatment could effectively inhibit the IL-1*β*-induced expression of inflammatory mediators, including NO and PGE2 [18]. Several studies found that the expression of IL-1*β*, NO, and PGE2 is significantly increased in arthritis tissue and OA animal models, even in patient serum [12, 19–22]. In addition to the local inflammatory mediators through the bloodstream, the essence of inflammation in OA involves the interplay of the innate immune system and inflammatory mediators, which cause chronic, comparatively low-grade, systemic inflammation [22]. This may be the most important reason for the increase in inflammatory mediators in serum. Combined with the results of this study, we found that FYC possesses anti-inflammatory effect in OA.

Articular cartilage also has some protection mechanisms in OA. TGF-*β* is a multifunctional cytokine involved in crucial biological processes, such as ECM synthesis, cell proliferation and differentiation, and tissue repair [23, 24]. Intra-articular injection of TGF-*β* increases proteoglycan synthesis and articular cartilage content in murine knee joints. Loss of TGF-*β* signaling in cartilage induces chondrocyte hypertrophy, which leads to cartilage degeneration, and activation of the TGF-*β* pathway has therefore been proposed to preserve articular cartilage integrity during OA [4]. HSP70 has been reported to be highly expressed in OA chondrocytes. It has the potential to prevent cartilage damage in arthritic joints and inhibit nitric oxide-induced apoptosis in chondrocytes by reducing caspase 3 activity [5]. Other studies further found that HSP70 promotes the expression of type II collagen and proteoglycan core protein in chondrocytes. RANKL, a member of the tumor necrosis factor family, can activate nuclear factor-*κβ* ligand through RANK–RANKL specific binding to stimulate osteoclast activation. OPG, a membrane-bound tumor necrosis factor-related factor, is an important cytokine that protects the cartilage and plays a vital role in regulating the biology of cartilaginous tissues [25]. It works as a decoy receptor for RANKL and therefore prevents RANK activation and subsequent osteoclastogenesis [6, 26]. The OPG/RANKL ratio is an index of osteoclastogenic stimulus. In this study, high levels of TGF-*β*, HSP70 and OPG/RANKL ratio were detected in the FYC group. Therefore, the protective role of FYC on articular cartilage was further confirmed.

## 5. Conclusion

Given that traditional Chinese medicine is composed of multiactive components and possesses multitarget action feature, it can aim at multiple targets, exerting a systemic effect at the same time. Multitarget medicine has been proven to be particularly effective in treating complex diseases [1, 27–29]. In this study, we found that FYC protected articular cartilage via numerous ways, including the reduction of ECM degradation factors, decrease in inflammatory mediator depression, and improvement of articular cartilage protective factor. FYC may be an effective agent for OA treatment.

The fact that FYC could protect the articular cartilage from injury by OA requires more studies. The useful compounds are still unknown, and a deeper insight into the deeper mechanism of FYC in OA should be provided. Several studies have indicated that nuclear magnetic resonance-based metabonomics analysis is an effective approach to assess the therapeutic effects of traditional Chinese medicine [30]. We look forward to do this work in the near future.

## Figures and Tables

**Table 1:** RT-PCR primers.

Gene	Sequence
ADAMTS-4	
Sense	5′GCCTTTCTCTGGTTTGGAGC3′
Antisense	5′GGCTGGTAATCGGTACAGCA3′
ADAMTS-5	
Sense	5′TTCCGTCATTAGCCACAGCAG3′
Antisense	5′GTGTCACAGGTCCTAGAGCAG3′
MMP-1	
Sense	5′ACAGTCCATGGATCCAGGTTATC3′
Antisense	5′CGGAGGCTAAATCTGCGTTG3′
MMP-2	
Sense	5′GCCCCCATGAAGCCTTGTTT3′
Antisense	5′GCTGGTGCAGCTCTCATACT3′
MMP-3	
Sense	5′TGTGGTTGTGTGCTCATCCT3′
Antisense	5′CCTGTCATCTCCAACCCGAG3′
MMP-9	
Sense	5′GAAAACCTCCAACCTCACGGA3′
Antisense	5′TTTGGAATCGACCCACGTCT3′
MMP-13	
Sense	5′TGAACATCCATCCCGTGACC3′
Antisense	5′ACTCCACACGTGGTTCTCAG3′
IL-1*β*	
Sense	5′ATGCCACCTTTTGACAGTGATG3′
Antisense	5′AGCTTCTCCACAGCCACAAT3′
OPG	
Sense	5′GCCACGCAAAAGTGTGGAAT3′
Antisense	5′TTTGGTCCCAGGCAAACTGT3′
RANKL	
Sense	5′TCGGGAAGCGTACCTACAGA3′
Antisense	5′CCCCAAAGTACGTCGCATCT3′
TGF-*β*1	
Sense	5′AGCTGCGCTTGCAGAGATTA3′
Antisense	5′AGCCCTGTATTCCGTCTCCT3′
GAPDH	
Sense	5′TCAGGAGAGTGTTTCCTCGT3′
Antisense	5′TGCCGTGAGTGGAGTCATAC3′
